# An EEG-Based Investigation of the Effect of Perceived Observation on Visual Memory in Virtual Environments

**DOI:** 10.3390/brainsci12020269

**Published:** 2022-02-15

**Authors:** Michael Darfler, Jesus G. Cruz-Garza, Saleh Kalantari

**Affiliations:** Department of Human Centered Design, Cornell University, Ithaca, NY 14850, USA; mbd25@cornell.edu (M.D.); jgc243@cornell.edu (J.G.C.-G.)

**Keywords:** social facilitation and inhibition, visual working memory, virtual reality, spatial environments, attentional focus, neurophysiology

## Abstract

The presence of external observers has been shown to affect performance on cognitive tasks, but the parameters of this impact for different types of tasks and the underlying neural dynamics are less understood. The current study examined the behavioral and brain activity effects of perceived observation on participants’ visual working memory (VWM) in a virtual reality (VR) classroom setting, using the task format as a moderating variable. Participants (*n* = 21) were equipped with a 57-channel EEG cap, and neural data were collected as they completed two VWM tasks under two observation conditions (observed and not observed) in a within-subjects experimental design. The “observation” condition was operationalized through the addition of a static human avatar in the VR classroom. The avatar’s presence was associated with a significant effect on extending the task response time, but no effect was found on task accuracy. This outcome may have been due to a ceiling effect, as the mean participant task scores were quite high. EEG data analysis supported the behavioral findings by showing consistent differences between the no-observation and observation conditions for one of the VWM tasks only. These neural differences were identified in the dorsolateral prefrontal cortex (dlPFC) and the occipital cortex (OC) regions, with higher theta-band activity occurring in the dlPFC during stimulus encoding and in the OC during response selection when the “observing” avatar was present. These findings provide evidence that perceived observation can inhibit performance during visual tasks by altering attentional focus, even in virtual contexts.

## 1. Introduction

Research on social facilitation and inhibition (SFI) effects has consistently shown that the presence of other people can alter individual performance and that the direction of the effect may depend on complex factors, such as individual working memory capacity [1], attitudinal and motivational orientation [2,3], observer attentiveness [4], personal implication of the activities [5] and task complexity [6,7,8]. While these effects have been well studied in both laboratory and field environments, far less is known about how individuals respond to perceptions of being observed in virtual environments.

### 1.1. Task Performance and Attentional Focus

There is a substantial body of research showing that the presence of external observers can have an effect on task performance. The direction of the effect (positive or negative) appears to depend on the type of task that is being carried out. Baron [9] theorized that “social presence” affects task performance by altering attentional focus and information processing. While earlier researchers hypothesized that the presence of other people in an environment increased drive and domination responses [10], Baron’s work indicated that the effect is better understood as a narrowing of attentional focus. For a simple task, this attentional focusing would logically help in tuning out peripheral and distracting information, thus increasing performance. However, for a more complex task that requires distributed attention and creative synthesis of a wide range of inputs, this attentional focusing effect of social presence would logically tend to decrease performance [9].

Later work by Huguet and colleagues [4] provided empirical evidence for some aspects of Baron’s theory by studying task performance under a variety of social presence conditions. This research used the Stroop test in which individuals are presented with words that spell out different colors, while the actual color of the font may either agree or disagree with the text (e.g., the word “green” spelled out in a blue font). Participants are asked to state the actual color of the font while ignoring what the characters of the word spell out. Response time (RT) for incongruent word/color combinations is compared against RT for neutral or congruent word/color combinations. The difference between these RTs is called the “Stroop interference,” and lower levels of Stroop interference are associated with an increase in focused task performance. Huguet and colleagues found that social presence was correlated with a decrease in the Stroop interference level, and that more judgmental social attention (going from an inattentive observer to a critical observer) reduced the Stroop interference even further. Thus, the effect of perceived judgmental observation was to improve performance on this simple/focused task. Huguet and colleagues interpreted these results to indicate that when under observation, participants are more likely to tune out extraneous information (the textual meaning) and focus on the prescribed task (identifying the color). A similar study by Klauer and colleagues [11] obtained congruent results, and likewise attributed the decreased Stroop interference under observation conditions as resulting from more focused attention.

### 1.2. Attentional Focus and Working Memory

Researchers have associated the phenomenon of attentional focus with the cognitive processes of working memory and executive functioning [12]. These are closely related concepts, and in fact, some researchers consider working memory as a type of executive function [13,14]. They each refer to the ability to maintain attention on short-term memory items in order to preserve them from distraction and prevent them from being forgotten [15,16]. Distractions and increased demands for attentional resources place a greater load on working memory [17], which in turn, threatens the maintenance of stored memory items and can lead to reduced performance on simple, focused tasks [12].

Visual working memory (VWM), the ability to retain a memory of visual images for a short period of time, is an important subset of working memory and is the fundamental basis of the brain’s “coherent and continuous representation of the external world” [18]. Similar to other forms of working memory, social observation has been shown to require attentional focus be paid to the observer, creating an increased performance effect for simple/focused visual tasks but a decreased performance effect for broader synthesis and analysis tasks. For example, in a study where participants were asked to examine baggage X-ray security images, the presence of observers was associated with decreased RT on simple reviewing tasks and increased RT on complex analysis tasks [19]. Visual working memory has been shown to be susceptible to distractions, especially those that share significant overlap with the target stimulus [20,21]. In particular, eye contact with another human has been shown to be distracting in a variety of VWM-based tasks, including simple visual target detection [22] and spatial cognition [23].

### 1.3. Neural Dynamics of Visual Working Memory

Studies in neuroimaging have identified three cortical regions that are critical to VWM: the occipital cortex (OC), the posterior parietal cortex (PPC), and the prefrontal cortex (PFC) (for reviews, see [18,24]). The sensory regions of the brain, including the OC, are thought to receive and encode sensory information (in this case, visual information) prior to storage in working memory. Once the representations are stored, working memory tends to be rather robust to outside distractors [20]. However, distractors that are displayed concurrently or in close temporal proximity (<50 ms) to the sensory information of interest can significantly impair visual working memory recall [25]. The PPC and PFC regions have both been implicated in VWM storage, with the latter region also implicated in the control of attentional resources and retrieval [26,27]. The PFC “communicates with sensory regions via top-down control during the delay period to facilitate upcoming comparisons” [18]. Several EEG studies have found that PFC theta-band dynamics plays an important role in the control of working memory [28,29]. Itthipuripat and colleagues [30] showed that increased theta-band power in this region was associated with successful working memory manipulations. Increased midfrontal theta-band activity was also observed when preparing to switch between two focused tasks [31,32].

Other frequency range dynamics, including alpha-band and beta-band activity, have also been experimentally associated with VWM. When faced with highly salient distractors during a visual search task, participants showed higher bilateral parieto-occipital alpha-band power prior to the presentation of the stimulus as well as during the search phase, suggesting the importance of alpha-band dynamics in the suppression of task-irrelevant information [33].

### 1.4. The Present Study

In the present study, the researchers examined the effect that perceived observation had on neurological VWM function and task performance in a virtual reality (VR) immersive environment, where a university classroom was 3D scanned for the setting. To the best of our knowledge, this is the first study to evaluate these phenomena in VR. The context of being “virtually observed” is interesting in the sense that there is a lack of empirical data about whether or not the resulting behavioral and neurophysiological effects will be commensurate with prior studies involving the physical, real-world presence of an observer. This is an important environment to study, as the use of virtual reality is rapidly increasing in contexts such as education, business, design, social interactions, and therapy [34,35,36]. Particularly in the context of education, VR approaches have been shown to enhance learning motivation, enjoyment, and situational interest in many student populations [34,37,38,39,40]. In the COVID-19 era, there has been an expanding interest in improving the fidelity of educational VR environments and learning more about how the social and environmental design of these environments can affect student learning.

Based on the previous literature discussed above, the study was designed with attentional focus theory in mind [9]. Accordingly, two vision-based tasks were used, which focused more on integration, processing, and actively reconstructing a visual pattern [41]. If the attentional focus theory is accurate and relevant in the VR context, then we would expect these effects to be evident in EEG neuroimaging data for the OC, PPC, and PFC brain regions. This study specifically examined the role of the OC and dlPFC. Analysis of the PPC was excluded due to insufficient data in that brain region from equivalent independent component dipoles.

## 2. Materials and Methods

The study protocol was evaluated and approved by the Institutional Review Board at the University of Houston, and all participants provided written informed consent to participate in the study. Informed consent for the publication of identifying images in an online publication was obtained for the study. All experiments were performed in accordance with relevant guidelines and regulations.

**Figure 1:**
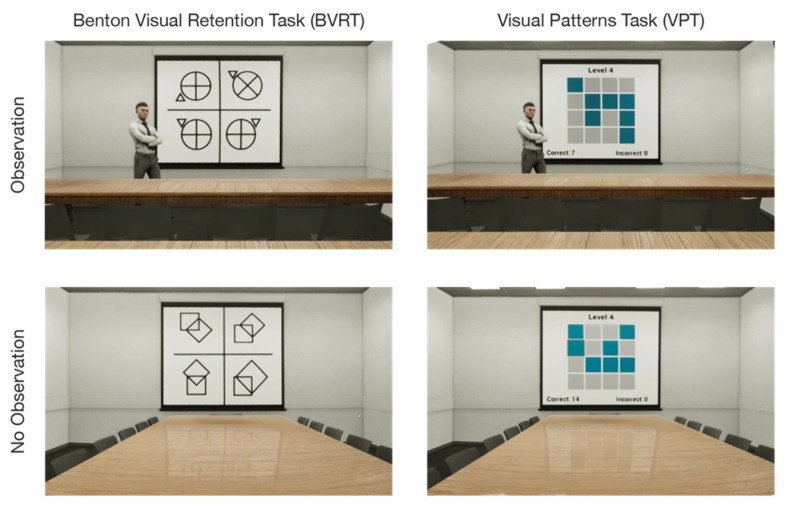
The study observation conditions (observation vs. no observation) with Benton visual retention task (BVRT) and visual patterns task (VPT). Observation was operationalized with the addition of a static human avatar at the front of the room.

The study used two WVM tasks, the Benton visual retention task (BVRT) and the visual patterns task (VPT). Each of these tasks were conducted under two different observation conditions, with and without (Figure 1). For the visual patterns task (VPT), which was previously used as a measurement of short-term visual memory storage and recall [42,43], participants were shown a grid of squares, either 3 × 3 or 4 × 4, where half of the squares (rounded up) in the grid are randomly selected to be presented in a different color from the others. Participants were asked to remember the pattern of colored squares and then reproduce them on a blank grid. As the grid size increases, so does the number of squares that must be stored in memory.

For the BVRT [44,45], the general procedure involves the presentation of a complex geometric figure that the participant must remember and later recall or reproduce. The version that was used in this experiment was similar to the “M-type” administration of the BVRT in that for the recall portion of the test, the participants were presented a grid of four rotated and altered designs and were asked to select the correct design that had not been altered [46]. According to Moses [47], this version of the BVRT (M-type) requires “complex perceptual analytic ability… [as well as] short-term memory retention” (p. 154).

The observation conditions were operationalized by the presence or absence of a static avatar standing just to the left of the screen on which the tests were projected. The participants were given no explanation regarding the presence or absence of this figure. The reason for omitting such an explanation was to reduce confounding variables related to the interpretation of the figure’s intent. Prior studies have shown that there may be complex interactions between stated observer intent and participant demographics, with diverse participants from varied backgrounds responding in very different ways to the same observation intent. While such divergences in participant responses cannot be fully avoided and may also be derived in part from the physical appearance or posture of the observer, the effect tends to be exacerbated when the purpose of an observer’s presence is explained [4,48,49,50]. Thus, providing the participants with no information at all about the observing avatar’s presence was intended to dampen the greater divergence in responses that may have occurred if we had given a “cover story” or indication of why the observer was present.

### 2.1. Participants

Twenty-nine healthy human adults with normal or corrected-to-normal vision were recruited for the study, using a convenience sampling method (word-of-mouth and announcements on departmental e-mail lists). Five of the participants had to be excluded due to technical issues during the physiological data-recording (missing/incomplete data), two additional participants were excluded due to extremely low task accuracy scores (<10%) across all conditions, and one participant was excluded due to video artifacts that impaired the researcher’s ability to accurately code behavioral data. The remaining 21 participants ranged in age from 18 to 55 years (M = 26.17, SD = 10.4). In regard to their gender, 8 of the participants identified as female, 13 identified as male, and none identified as other. Ten of the participants reported as Asian, six as Latinx and/or Hispanic, one as Middle Eastern, and four as White non-Hispanic.

An a priori analysis of the required sample size was conducted using G*Power (Version 3.1.9.3.). An original minimum sample size of 27 participants was estimated for a 0.05 probability of error, a power of 0.80, and a projected effect size of dz = 0.55. Due to the exclusion of eight participants as a result of technical glitches, we did not achieve the projected minimum sample size of 27. However, our existing sample size of 21 participants is viable for a 0.05 probability of error, a power of 0.80, and an effect size of dz = 0.66. The assumption of the effect size is consistent with the results of similar prior EEG studies, which have generally found a dz between 0.60 and 0.80 [34,51,52,53].

### 2.2. Virtual Reality Development

The virtual environment was developed and presented to participants using Epic Games’ Unreal Engine (www.epicgames.com, accessed on 28 October 2021). It was based on a seminar-classroom setting and was only slightly altered between the different experimental conditions. A widget was used to display the two different cognitive tests on a virtual projector-screen at the front of the classroom, and a static avatar along with slight furniture alterations were added for the observation conditions (Figure 1). The experience was presented to the research participants using an Oculus Rift head-mounted display with a resolution of 1920 × 1080 pixels (960 × 1080 pixels per eye). The refresh rate was set at 72 Hz. Participants were seated in a chair but could look around the virtual environment by moving their heads. The viewing position within the room was fixed in order to maintain a consistent field of view for each participant.

While previous studies have associated increased avatar realism in virtual environments with an increased sense of immersion and social presence, researchers have also found that individuals are able to adapt and ignore relatively high levels of digital abstraction [54,55,56,57]. The VR presentation in the current study made use of the best commercially available technology to enhance the realism of the environment. Previous work on behavioral and neurological responses in such VR environments have shown that the high-immersive and realistic VR experiences can be used as a reliable proxy for physical environments [58,59,60,61]. In a previous study from our research group, participants completed a variety of cognitive tasks in a real classroom and in a virtual reproduction of the same classroom, and the EEG data did not show significant differences in frequency band-power between the real and virtual conditions [51].

### 2.3. Data Collection

Behavioral data were collected in the form of controller signals and screen recordings. The participants used a handheld Xbox controller to respond to test questions. The joystick on this controller was used to toggle between multiple-choice answers, while various buttons were used to select answers and advance through the tests. These signals were all logged for later analysis. A high-definition screen recording was made of what each participant saw as they looked around the virtual environment, thus helping to track the participants’ visual attention and verify response accuracy and timing. Neurological activity was measured using a 63-channel actiCHamp system (Brain Products GmbH, Gilching, Germany). Fifty-seven electrodes were devoted to scalp EEG recordings, an additional four electrodes were devoted to EOG data (two placed laterally 1 cm outside the eyes, and two vertically from the right eye), and the final two electrodes were devoted to ECG measurement [62]. The BrainVision Recorder software package was used for all electrophysiological data recording. The current analysis involves only the 57 EEG scalp electrodes and the task-performance data.

### 2.4. Experimental Procedure

Sessions were conducted for one participant at a time. Upon arrival, each participant was provided with an introduction to the study procedure and was then asked to give written consent to the study procedure. After giving consent and completing an initial intake survey, each participant was fitted with an EEG cap and a VR headset and was given a few minutes to explore the virtual environment in order to become familiar with the controls. Baseline EEG recordings were taken by asking the participant to focus on a static image (fixation cross) for 1 min and then to close their eyes for one minute. The participant was then given a ten-minute period to become familiar with the cognitive tests and to practice the input methods for submitting test answers.

**Figure 2:**
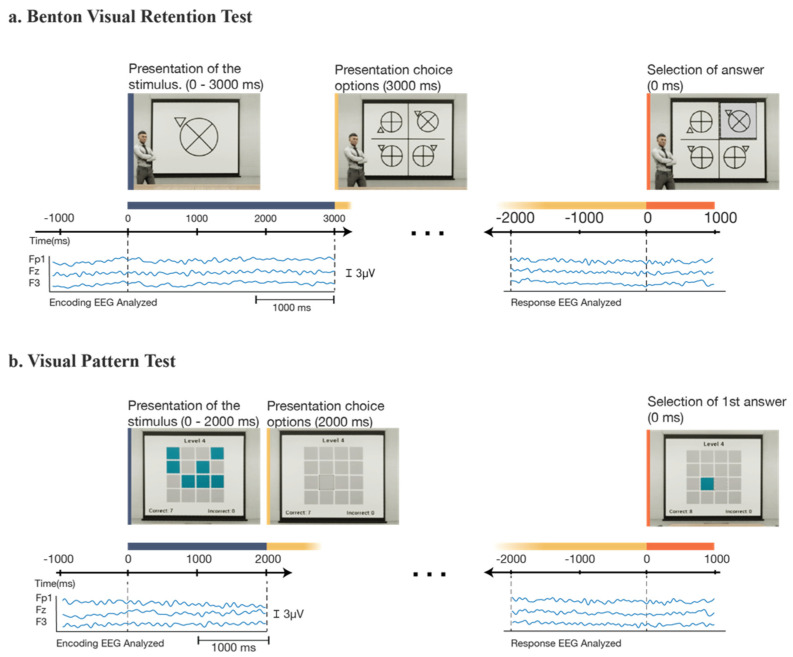
An overview of the experimental procedure. Behavioral responses and EEG data were collected for each participant in each of the four experimental conditions, starting with (**a**) the Benton test (under observed and non-observed conditions), and then continuing to (**b**) the visual patterns test (under observed and non-observed conditions). For each condition, participants completed 4 trials for a total of 16 trials. After each condition, participants indicated their stress and mental fatigue. In between tasks (BVRT and VPT), participants were given a 1 min rest.

For the evaluation part of the study (Figure 2), each participant experienced the four conditions in a random order. In the BVRT, the participant was shown a stimulus figure for 3 s. Immediately after this, a grid of four options was presented, and the participant was asked to select the figure that they believed was the same as the stimulus figure. There was no time limit for the answer, and no feedback was given about correct or incorrect answers. This test was repeated four times under the same observation condition, after which the participant indicated their stress and mental fatigue levels on a scale of 1 (no stress/fatigue) to 10 (the most stress/fatigue).

The same procedure was performed for the VPT, where a grid of randomly colored and uncolored squares was shown for 3 s. Immediately after this, the participant was shown a blank grid upon which they were asked to recreate the stimulus pattern. Incorrect answers were indicated to the participant on this test, and the trial continued until the participant had correctly identified all of the colored squares from the stimulus. Four trials were completed in each observation condition. The first two VPT trials showed a 3 × 3 grid with five colored squares, and the third and fourth trials showed a 4 × 4 grid with eight colored squares. The test was then repeated in the other observation condition. Fatigue and stress levels were reported after each observation condition. Accuracy was assessed by the number of correct answers divided by the total (correct and incorrect) answers. The participants were given a 60 s rest after each trial, followed by a brief baseline period. After finishing four conditions, the EEG cap and VR headset were then removed from the participant’s head, and participants were asked to fill a brief exit survey about their VR experience. All participants were given a USD 25 gift card after completing the experiment.

### 2.5. Data Processing and Analysis

The behavioral data (test performance outcomes) were processed by analyzing the screen recording, hand coding the relevant time points and answer accuracy of each participant, and matching responses with controller signals in order to correlate the behavioral responses with the neurological data. For each test, in each observation condition, the start and stop times from the screen recording were recorded and quantized to the nearest second in order to obtain the total test duration. Because each participant was tested in all four conditions in a within-subjects design, the test accuracy and duration were analyzed using one-way ANOVA with observation and task format as within-subjects factors and the subject as a random effect [63]. Satterthwaite’s method was used to approximate the degrees of freedom. The statistical analysis was conducted using the lme4 package inside of the R software environment [64].

The EEG data were pre-processed using the EEGLAB software package [65]. Raw .xdf data files were imported at their original sampling rate of 500 Hz. The data were band-pass filtered between 0.5 and 50 Hz using the EEGLAB function “pop_eegfiltnew” (FIR, Hamming windowed, transition bandwidth 0.5 Hz). Artifact subspace reconstruction (ASR) [66,67] was carried out through its EEGLAB implementation with the following parameters: flat line threshold of 10 s, line-noise threshold of 4 std, and correlation coefficient with adjacent channels of at least 0.75. ASR was also used to remove short-time high amplitude artifacts in the continuous data, with a cut-off threshold of 20 standard deviations for the reconstruction of corrupted subspaces. Removed channels were then reintroduced to the data, using spherical interpolation from neighboring channels. The data were common-average referenced. The data were then run through an independent component analysis using the extended Infomax algorithm [68], and dipole-fitting using the Dipfit package, with the source-space warped to fit a template MNI space [69]. Artifactual components representing eye-movements, head movements, and other gross non-brain components were removed automatically using the ICLabel EEGLAB plugin, with a label probability threshold of 0.90 for muscle, eye, heart, and channel-noise related artifacts [70,71].

From the preprocessed and labeled EEG data, an epoch was extracted, corresponding to each visual memory task and observation condition (four epochs per participant). The mean baseline value for each epoch was removed based on the 1000 ms preceding the time-locking event in order to better compare transformation in the data across participants. Following the baseline removal, two different sub-epochs were extracted from each of the test iterations to study separately the stimulus encoding and the stimulus response. The stimulus encoding epoch began 1000 ms prior to the presentation of the stimulus (the original evaluation image) and lasted until the stimulus was removed from the screen. The stimulus response epoch began 2000 ms prior to the first answer response on the test and ended 1000 ms after the first answer response. The extra time of the epoch either before the time locking event (presentation of the stimulus) or after (response selection) was used as the epoch baseline, respectively. The time-frequency analysis was performed with the “newtimef” function in EEGLAB. We specified a frequency range from 3 to 30 Hz in a logarithmic scale, using a Morlet wavelet transformation with 3 cycles for low frequency and 0.5 cycles for higher frequency.

After all of the EEG data epochs were extracted, the previously identified independent components (ICs) with a retained variance less than 25% were rejected, as were ICs that were located outside of the brain. The remaining ICs were used to calculate event-related spectral perturbations. These were used, along with dipole and scalp locations, to identify IC clusters using the “k-means” clustering algorithm [52]. Two clusters, one located in the dlPFC and one in the OC, were chosen for analysis based on the previous literature, which implicated these brain regions in the storage and retrieval of short-term visual memory [53,72,73]. The first cluster contained 25 ICs from 13 participants. Its centroid was located at [x = −7, y = 54, z = 40] MNI, which is in the left dorsolateral prefrontal cortex (dlPFC) Brodmann area 9. The second cluster contained 24 ICs from 14 participants. Its centroid was located at [x = 9, y = −93, z = 24] MNI, which is in the occipital cortex (OC) at Brodmann area 18.

The analysis of the EEG data was primarily based on event-related spectral perturbations (ERSPs) in the selected IC clusters in the dlPFC and OC brain regions. ERSPs can reveal aspects of event-related brain activity, across both time and frequency, which are not contained in other analyses, such as event-related potentials or spectral analysis plots [74]. In the current study, the ERSPs were calculated using EEGLAB, for each of the two observation conditions (observation and no observation), for each of the two visual tasks, for each of the data sub-epochs (stimulus–presentation epoch and answer–response epoch), for each of the two brain regions (dlPFC and OC). In total, 2 × 2 × 2 × 2 = 16 ERSPs were computed from the combined participant data. Comparisons of the ERSPs were made between the different observation conditions for each visual task (x) epoch (x) brain region, using a one-way repeated-measures ANOVA at a significance level of *p* < 0.05. For each of these comparisons, the ERSP from the no-observation condition was subtracted from the observation condition, and only statistically significant differences were plotted. This allowed us to obtain a plot of significant ERSP differences that retained the directionality of each difference.

## 3. Results

### 3.1. Behavioral Results

**Figure 3:**
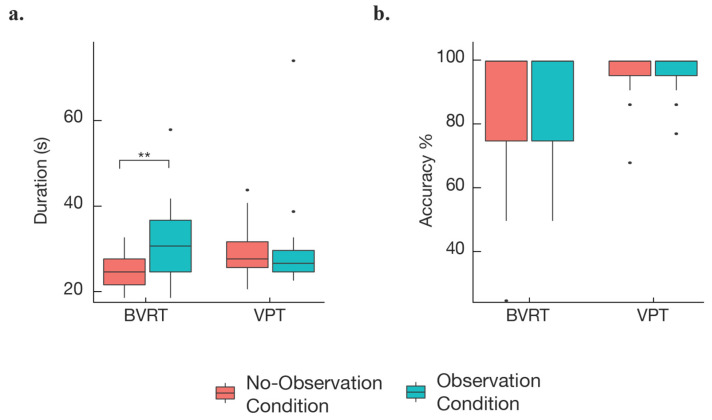
Overall behavioral findings: (**a**) the task-duration results indicated a highly significant effect of perceived observation for the BVRT task, but no significant effect of perceived observation for the VPT task. (**b**) No significant effects of perceived observation were found when analyzing task accuracy, though this may be due to a ceiling effect, particularly for the VPT task where the mean scores were very high at 96%. ** indicates *p* < 0.01.

A summary of the behavioral data is shown in Table 1 and Figure 3. Two types of behavioral results were analyzed in this study: task accuracy and task duration. The results of the task-accuracy analysis showed no effect of observation vs. no-observation conditions (F_1, 60_ = 0.0014, *p* = 0.97). However, results from the task-duration analysis (Table 2) did indicate a significant effect of observation vs. no-observation (F_1, 60_ = 7.11, *p* < 0.01). No significant effect of observation on task duration was found for the VPT (β = −0.714, t-ratio = −0.347, *p* = 0.7298.) However, on average, during the BVRT, individuals who were observed took 7.048 s (28%) longer to complete the four trials when compared to those who were not observed (Table 1).

These results provide partial support for the hypothesis that perceived observation impairs performance on visual memory tasks (in the sense that observation appeared to lengthen the time for task completion for the BVRT). There was no effect in the behavioral data for the VPT—though this latter outcome may have been due to a ceiling effect, since the mean accuracy of the participants on the simple task (VPT) was very high at 96%.

### 3.2. EEG Results

**Figure 4:**
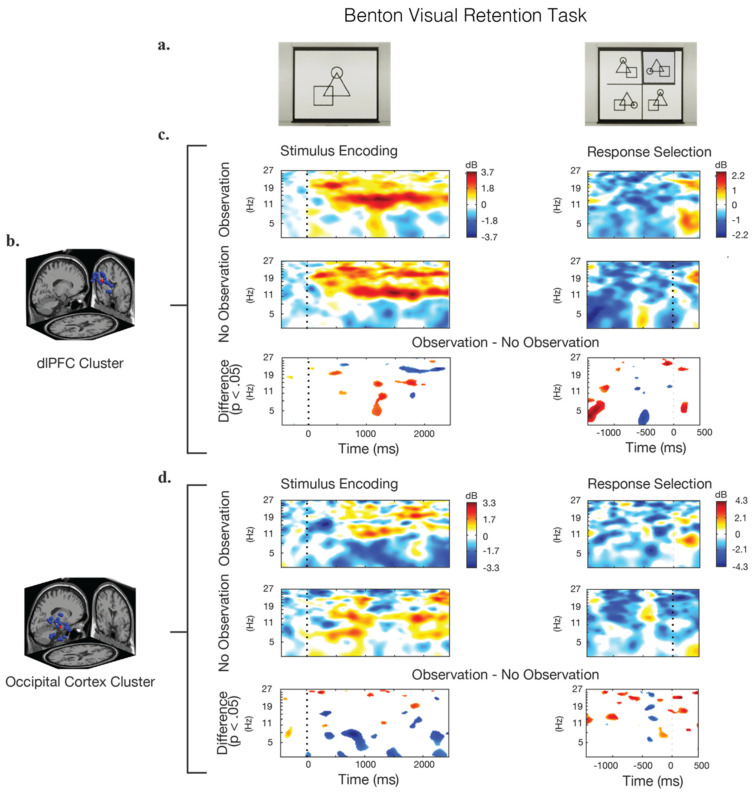
Event-related spectral perturbations (ERSP) during the stimulus presentation and response epochs for the Benton test. (**a**) Screenshot examples of the visual stimulus and response options. (**b**) The IC clusters selected for analysis were located in the dorsolateral prefrontal cortex (dlPFC) and the occipital cortex (OC); the locations of the individual IC dipoles are shown in blue, and the equivalent dipole location is shown in red. (**c**,**d**) Calculated ERSPs for the observation condition, the no-observation condition, and their significant differences. Frequency power is baselined to the beginning of the trail. Blue regions in the difference graphs indicate that the observation condition showed significantly lower power, while red regions indicate that the observation condition showed significantly higher power.

The analysis of ERSPs for the Benton task is shown in Figure 4. During the stimulus–presentation epoch of this task, the frontal cortex (dlPFC) showed strong and sustained activity in the alpha-band (8–12 Hz) across both the observation and no-observation conditions. There were significant differences during this epoch between the two room conditions, with the observation condition exhibiting higher alpha synchronization beginning approximately 1200 ms after the presentation of the stimulus. The dlPFC theta-band (4–7 Hz) activity in the observation condition likewise showed a large increase in power 1200 ms after the stimulus when compared to the no-observation condition. The trend was reversed when considering beta-band (12.5–30 Hz) activity, with the no-observation condition showing higher beta activation, especially after 1600 ms.

Considering the answer–response epoch of the Benton test, a significantly greater theta activation in the dlPFC occurs 500 ms prior to the selection of the response in the no-observation condition, compared to the observation condition. Significant theta-band differences between the conditions also appear at 1500 ms prior to the response, but the interpretation of this phenomenon is difficult due to its distal relationship to the response selection, and so its potential meaning is not discussed.

Activity in the OC cluster during the Benton test demonstrated lower theta-band power in the observation condition, especially between 600 and 1000 ms after the presentation of the stimulus. In both the observation and no-observation conditions, an alpha- and beta-band oscillation of around 1 Hz persists throughout the trail. During the response epoch, a broad-spectrum power increase occurs approximately 400 ms prior to the response in the no-observation condition. In the observation condition, this same power increase is present but significantly weaker, with a noticeable decrease in theta-band power also occurring during that time.

**Figure 5:**
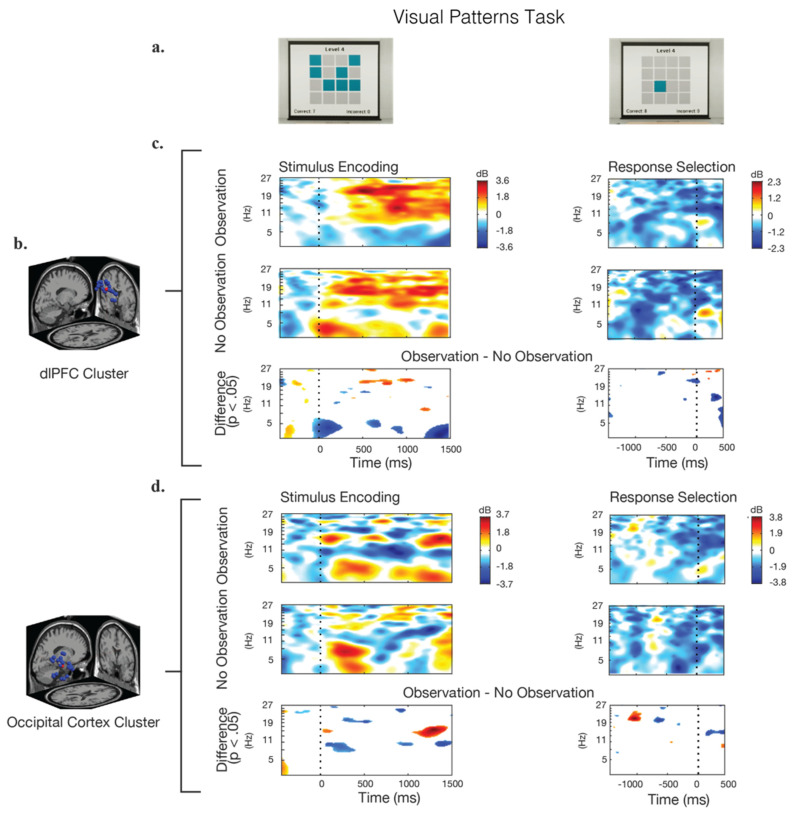
Event-related spectral perturbations (ERSP) during the stimulus presentation and response epochs for the visual pattern test. (**a**) Screenshot examples of the visual stimulus and response options. (**b**) The IC clusters selected for analysis were located in the dorsolateral prefrontal cortex (dlPFC) and the occipital cortex (OC); the locations of the individual IC dipoles are shown in blue and the equivalent dipole location is shown in red. (**c**,**d**) Calculated ERSPs for the observation condition, the no-observation condition, and their significant differences. Frequency power is baselined to the beginning of the trail. Blue regions in the difference-graphs indicate that the observation condition showed significantly lower power, while red regions indicate that the observation condition showed significantly higher power.

The analysis of ERSPs for the VPT is shown in Figure 5. In these data, similar patterns of alpha- and beta-band power synchronization, as in the Benton test (Figure 4) were seen in the dlPFC during the stimulus–encoding period for conditions observation, and no-observation. Theta-band activity in the dlPFC was significantly higher throughout the VPT in the no-observation condition, with a noticeable decrease in power at approximately 1100 ms after the stimulus presentation. There were no significant differences observed between the two observation conditions in the dlPFC in the time leading up to the response (response selection). Approximately 400 ms prior to the response, an increase in the theta and alpha band power was observed in both conditions.

Following the presentation of the stimulus, oscillatory patterns in the theta-, alpha-, and beta-bands were found in both observation conditions, though the effect was more pronounced in the observation condition. For the response time epoch, approximately 650 ms prior to the response selection, a broad-spectrum power increase occurred in the OC. This was more pronounced in the no-observation condition, though the difference was only significant in the beta-band. This pattern was similar to the one observed in the BVRT task, though it occurred approximately 250 ms earlier in the VPT condition. A strong alpha-band power increase was also seen in the observation condition in the OC 800–1800 ms after the presentation of the stimulus, which was significantly higher than the alpha-band activity in the no-observation condition.

### 3.3. EEG Theta-Band Results

**Figure 6:**
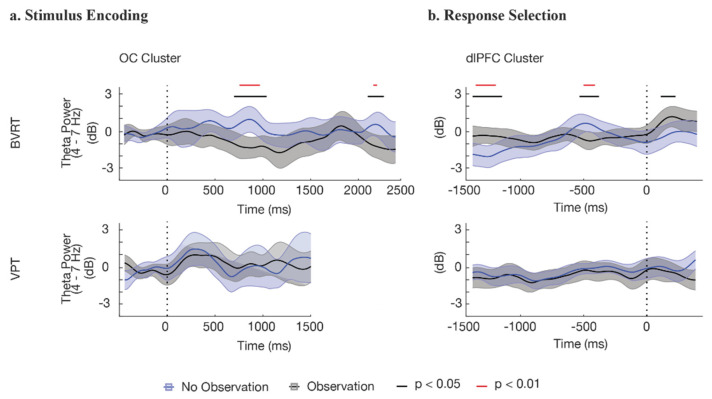
Theta-band (4–7 Hz) power for the observation and no-observation conditions, plotted with a 95% confidence interval. (**a**) during the stimulus period, the OC cluster showed significant power differences associated with observation conditions in the BVRT at approximately 725–1075 ms. No corresponding significant differences were observed in the VPT. (**b**) During the response period, the dlPFC cluster showed significant differences associated with observation conditions in the BVRT at approximately 400–500 ms prior to the response. No corresponding significant differences were observed in the VPT.

Prior literature has suggested that theta-band dynamics plays an important role in working memory and visual processing [53,72,73]. Therefore, an additional and more detailed analysis was conducted to explore the power dynamics of the theta-band over time, comparing the observation condition to the no-observation condition. Data from the ERSPs between 4 and 7 Hz were averaged over the frequency range for each task during the stimulus–presentation and response epochs. The analysis of this data confirmed significant differences in theta-band power between the two observation conditions in the BVRT, as well as a lack of such significant theta-band differences in the VPT task (Figure 6). In the BVRT, starting at 725 ms after the presentation of the stimulus, peaking at 900 ms, and ending at 1075 ms, the theta-band power in the no-obseravation condition was significantly higher than the observation condition (t_46_ = 3.54, *p* < 0.001, Cohen’s d = 1.02 at time = 900 ms). A similar theta-band power increase occurred in the no-observation condition in the dlPFC starting 550 ms prior to the response, peaking at 100 ms later, and ending after 175 ms, which was not seen in the observation condition (t_72_ = 2.94, *p* ≤ 0.005, Cohen’s d = 0.68). In the VPT task, there were no significant differences in theta-band power between the two observation conditions during the encoding period in the OC, or in the pre-response period in the dlPFC.

## 4. Discussion

It has been well documented that the physical presence of an observer or even the suggestion of an observer can impact task performance [1,7,8,9,10,50]. However, little research has been conducted about the effect of observation in a virtual environment, and we do not know much about the underlying neural dynamics associated with these behavioral outcomes. Using a theoretical perspective of attentional focus [9], this study examined how external observers (as operationalized by the presence or absence of a static human avatar) affect visual working memory (VWM) in virtual environments. This was accomplished using a within-subject experiment with two conditions of observation studies under two different VWM tasks. On average, when participants were observed, they took significantly longer to complete the BVRT task compared to the VPT, but the task accuracy was unaffected. Analysis of EEG data showed that the presence of an “observing” avatar during the BVRT was associated with increased theta-band activity in the occipital cortex (OC) following the presentation of the stimulus as well as in the dorsolateral prefrontal cortex (dlPFC) prior to the response selection, in comparison to the no-observation condition. There was no statistically different theta-band activity between the observation and no-observation conditions during the VPT recall task.

These findings are generally in line with previous literature on the effects of perceived observation for task performance, while extending those prior findings to show that they continue to hold, even when the “observer” is a motionless avatar in a virtual environment. The lack of significant differences in task accuracy should not be interpreted as evidence countering previous findings, given the likely presence of task-accuracy ceiling effects. These findings, along with the lack of significant EEG differences between the observation vs. no-observation states in the VPT task, may also have been affected by the low number of trials that were conducted (only four per participant for each condition). Future studies may produce more nuanced results by evaluating a wider array of more complex tasks and conducting a greater number of trials. The current study also used a limited sample size and a convenience sampling method, with demographics that leaned toward men in their twenties. Future studies with a larger and more diverse set of participants could enhance the strength of the study results as well as our understanding of individual differences in the effects of perceived observation on visual working memory.

In regard to the EEG data, brain dynamics in the occipital cortex (OC) and the dorsolateral prefrontal cortex (dlPFC) were analyzed. Both of these regions have previously been implicated in the functioning of visual working memory [18,28,30,75]. Results of this study’s analysis showed consistent and identifiable changes in brain dynamics across the alpha, beta, and theta frequency bands during the visual stimulus presentation and test–response periods. The EEG results suggest that theta-band dynamics in these brain regions are susceptible to the presence of a “virtual observer.” The presence of the observer was associated with the suppression of theta-band power during storage and retrieval of visual information, but only in the BVRT. Previous EEG-based studies have associated increased theta-band dynamics with higher mental workload and with task-switching [29,33,75]. Therefore, these EEG results appear to support the theory that the presence of an observer alters task performance by affecting attentional focus [9]. In the BVRT, prior to the task response, individuals in the observation condition generally showed higher theta activity, though not significantly so, in the dlPFC, which may suggest a higher overall workload caused by the requirement to attend to both the observer and the visual memory task. However, immediately prior to the selection of the response, the increase in theta-band power was found in the no-observation condition that was not present for those under observation. This may suggest a weaker task-switching response, i.e., due to the increased attentional demands that were paid to the observer compared to VPT, participants were not as effectively able to switch from encoding and storage to retrieval and response [75].

Observation in this study was operationalized through the use of a static human avatar facing toward the participant. This narrow operationalization should be expanded on in several important ways. Previous studies have shown that one of the most important visual stimuli that we perceive are other people’s eyes and that we closely attend to where others are looking, thus consuming critical attentional resources [76]. However, by using mouth shapes that indicated observer directionality, Colombatto et al. [77] showed that the disruption of working memory due to external observers can be achieved without any attention to the observer’s eyes and may reflect, what the authors called, “mind contact.” These finding suggests the need to explore different ways of indicating observational attention to the observed and whether the directional focus of the observer modifies the effect on task performance.

Furthermore, within the realm of VR, representational fidelity plays an important role in behaviors and can alter perceptions of immersion [55], object location memory [57,78,79], quality of social interaction [57] and embodiment [56]. Garau et al. [54] showed that increased avatar realism and eye-gaze control increased individuals report of the quality of the interaction, and that with low fidelity, avatars’ improved gaze control can increase reports of negative interactions. This suggests the importance of the match between the visual and behavioral realism of an avatar. Weyers et al. [80] similarly investigated responses to static and dynamic facial expressions of virtual avatars and found that dynamic expressions lead to increased reports of intensity and realism as well as greater activation of emotion-specific facial muscular activation. Future studies should investigate how the visual and behavioral dynamics of avatars in the virtual environment affect individual performance and attention.

## 5. Conclusions

This study addressed the behavioral and neurophysiological effects of observation on visual working memory and its interaction with the task format. The findings confirmed that observation can negatively affect response times on visual working memory tasks and expanded on those findings by replicating these results in a virtual environment with a static avatar. Consistent, identifiable changes in EEG dynamics in the occipital cortex and the dorsolateral prefrontal cortex were also found in the observation vs. the no-observation conditions—specifically, increases in theta-band (4–7 Hz) dynamics in the OC following the visual stimulus presentation and in the dlPFC prior to task responses. These findings suggest that the increased attentional demands caused by the observation interfere with both the encoding and retrieval of visual memory.

## Figures and Tables

**Table 1:** Summary of behavioral data with means and standard deviations of task duration and accuracy for each of the two experimental conditions as well as by task format condition and observational condition.

	Duration		Accuracy	
	Mean (s)	SD (s)	Mean	SD
BVRT	28.8	7.78	0.857	0.19
Observation	32.3	8.95	0.86	0.22
No-Observation	25.3	4.24	0.86	0.17
VPT	29.6	8.49	0.96	0.07
Observation	29.9	10.8	0.99	0.06
No-Observation	29.2	5.58	0.96	0.07
Observation	31.1	9.87	0.91	0.14
No-Observation	27.2	5.28	0.91	0.17

**Table 2:** Type III analysis of variance table with Satterthwaite’s method of condition on duration. A significant difference between task durations was observed when comparing participants who were observed with those that were not observed. No significant effect of task format was observed. ** indicates *p* < 0.01. Definitions. Sum of Sq: Sum of Squares. Mean sq: Mean Squares, NumDF: Numerator, Degrees of Freedom. DenDF: Denominator, Degrees of Freedom.

	Sum Sq.	Mean Sq.	NumDF	DenDF	F Value	Pr (>F)	
Observation vs. No Observation	316.3	316.3	1	60	7.11	<0.010	**

## Data Availability

The data presented in this study are available on request from the corresponding author.
